# Recent Advances of Metal–Polyphenol Coordination Polymers for Biomedical Applications

**DOI:** 10.3390/bios13080776

**Published:** 2023-07-31

**Authors:** Jing Qin, Ningning Guo, Jia Yang, Yong Chen

**Affiliations:** College of Advanced Materials Engineering, Jiaxing Nanhu University, Jiaxing 314001, China; 19518318273@163.com (N.G.); y19557377107@163.com (J.Y.); cy11437758@163.com (Y.C.)

**Keywords:** polyphenol, metal–phenolic coordination polymers, biomedical, tumor therapy, antibacterial

## Abstract

Nanomedicine has provided cutting-edge technologies and innovative methods for modern biomedical research, offering unprecedented opportunities to tackle crucial biomedical issues. Nanomaterials with unique structures and properties can integrate multiple functions to achieve more precise diagnosis and treatment, making up for the shortcomings of traditional treatment methods. Among them, metal–polyphenol coordination polymers (MPCPs), composed of metal ions and phenolic ligands, are considered as ideal nanoplatforms for disease diagnosis and treatment. Recently, MPCPs have been extensively investigated in the field of biomedicine due to their facile synthesis, adjustable structures, and excellent biocompatibility, as well as pH-responsiveness. In this review, the classification of various MPCPs and their fabrication strategies are firstly summarized. Then, their significant achievements in the biomedical field such as biosensing, drug delivery, bioimaging, tumor therapy, and antibacterial applications are highlighted. Finally, the main limitations and outlooks regarding MPCPs are discussed.

## 1. Introduction

In recent years, the rapid development of nanomedicine has provided new technologies and methods for modern biomedical research, offering novel perspectives to address important biomedical problems at the nanoscale [1,2,3,4,5]. Nanomaterials with unique structures and properties have significant advantages for biomedical applications. The surface of nanomaterials can be functionalized, giving them excellent properties such as biocompatibility, environmental responsiveness, and specific targeting ability [2]. Additionally, nanoplatforms have unique pharmacokinetic and biological distribution characteristics, which are conducive to the enrichment of focal areas and achieve superior imaging performance and therapeutic effect [3]. Moreover, the nanocarriers can improve the stability of small molecule drugs, dispersion and absorption utilization ratio, and reduce the side effects of drugs [4,5].

**Figure 1:**
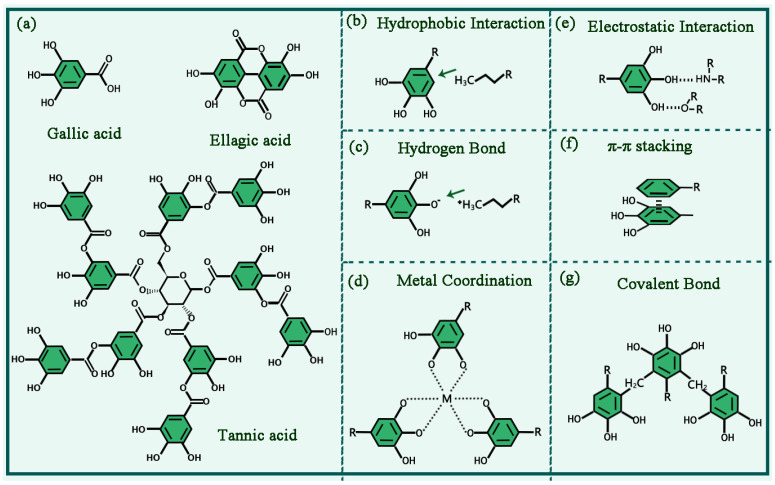
(**a**) The chemical structure of several plant polyphenols. (**b**–**g**) Covalent interactions and noncovalent interactions of plant polyphenols.

Polyphenols, a kind of natural organic compounds, belong to plant secondary metabolites and exist commonly in vegetables, tea, and other plants [3,4,5]. Polyphenols have strong adhesion and antioxidative ability, which can resist ultraviolet rays and clear against free radicals. In addition, polyphenols have been reported to show anti-inflammatory, antiaging, and anticancer properties [5,6]. The polyphenol structure contains two or more phenolic hydroxyl units connected by stable C-C or ester bonds [7,8,9]. Polyphenols can be divided into three major categories according to their chemical structure: dihydroxyphenol (ellagic acid, quercetin, etc.), trihydroxyphenol (gallic acid, pyrogallol, baicalein, etc.), and dihydroxyphenol and trihydroxyphenol mixed systems (tannic acid, epicatechin gallate, etc.) (Figure 1a) [5]. Due to the presence of phenolic hydroxyl groups and benzene rings, polyphenols can be assembled using different metal ions, molecules, and substrates through covalent interactions (Michael addition, Schiff base reactions, and coordination interactions) and noncovalent interactions (hydrogen bonds, π-π stacking, and electrostatic interaction) (Figure 1b–g) [10,11,12,13,14]. In recent years, polyphenols have been widely used as excellent raw materials for the preparation of multifunctional nanomaterials, and have broad application prospects in the fields of energy, environment, and biomedicine.

Metal–polyphenol coordination polymers (MPCPs) are assembled via the coordination of metal ions and phenolic ligands. Metal ions display a diverse array of electronic, optical, radioactive, magnetic, and catalytic properties, rendering MPCPs as exceptional nanoplatforms for imaging and therapy [15]. MPCPs have been extensively investigated in the field of biomedicine owing to the advantages as follows: (i) The fabrication process of MPCPs is simple and environmentally friendly [16]. (ii) MPCPs can be readily functionalized to construct multifunctional nanoplatforms, thereby enhancing the therapeutic efficacy of diseases [17,18]. (iii) The pH-responsiveness of the MPCPs facilitates targeted drug delivery and controlled release, allowing for precise treatment of diseases. (iv) MPCPs demonstrate excellent biocompatibility and negligible toxicity [19,20].

**Figure 2:**
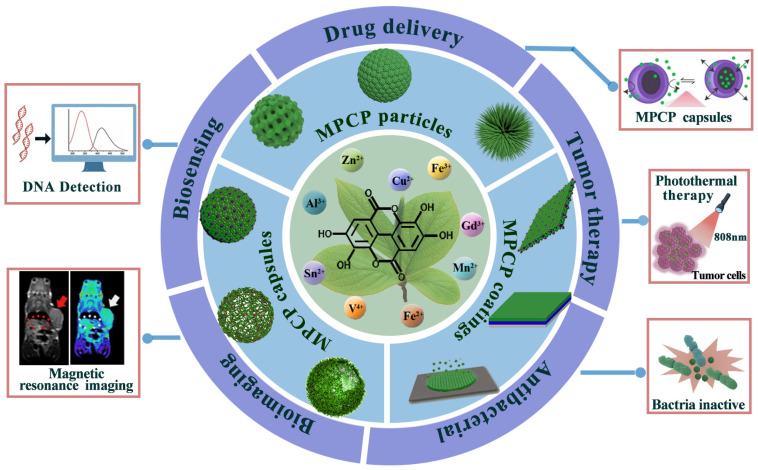
The review of various MPCPs structures for biomedical applications.

Recently, some reviews regarding MPCPs for cancer theranostics have been reported. For instance, Xie et al. summarized the self-assembly methods and shape-controllable preparations of MPCP systems, while emphasizing the remarkable progress achieved in various MPCPs for cancer theranostics [18]. Meanwhile, the overview of the properties, fabrication methods, and applications in cancer theranostics of MPCPs was provided by Zhang et al. [19]. However, there is a lack of comprehensive reports on the application of MPCPs in drug delivery, antibacterial and biosensing, beyond cancer theranostics. Therefore, this review aims to provide a thorough examination of recent advances in MPCPs for biomedical applications such as biosensing, drug delivery, bioimaging, tumor therapy, and antibacterial applications (Figure 2). We commence by presenting the categorizations and fabrication strategies of diverse MPCPs systems (particles, capsules, and coatings). Then, the significant achievements in the biomedical field were highlighted. Finally, we discuss the main limitations and potential opportunities encountered by MPCPs.

## 2. Synthesis of Metal–Polyphenols Coordination Polymers

Metal–polyphenol coordination polymers (MPCPs) are supramolecular structures composed of metal ions and polyphenol ligands. The abundant phenol hydroxyl groups in polyphenols can form five-sided ring structures via coordination with different metal ions. The coordination processes are not only dependent on polyphenols and metals ions species but are also mainly regulated by pH [15,16,17,18,19]. The phenol hydroxyl groups expose the oxygen center with high charge density after deprotonation under alkaline conditions, which is favorable for coordination assembly with metal ions [20]. The assembly of MPCPs does not require special solvents or external heating, electricity or light, and the preparation process is facile and environmentally friendly. Metal ions and polyphenol ligands can be assembled into MPCPS with various structures, such as particles, capsules, and coatings [20]. In this part, different MPCPs systems will be discussed. There are representative MPCPs listed in Table 1.

### 2.1. MPCP Particles

Polyphenols and metal ions can be rapidly assembled to form metal–polyphenol coordination particles. Common MPCP particles include nanoparticles, crystal particles, and mesoporous particles.

#### 2.1.1. MPCP Nanoparticles

**Figure 3:**
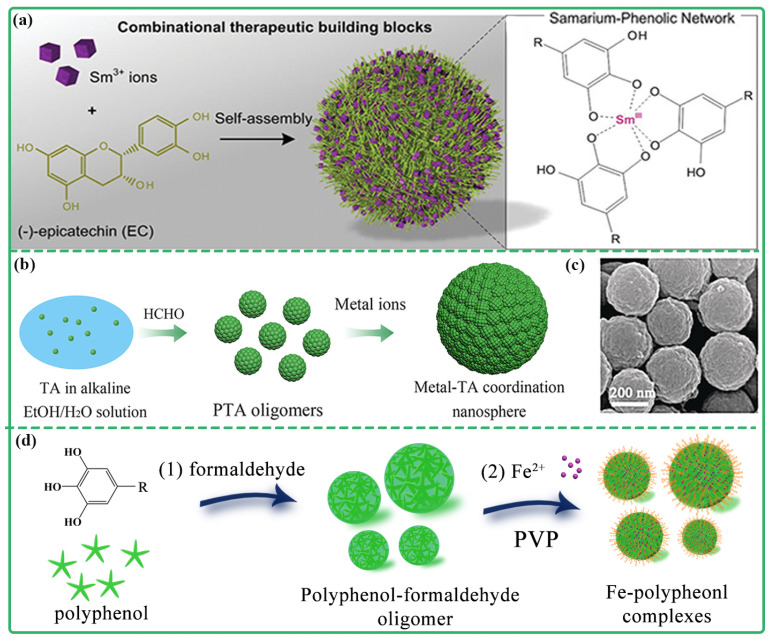
(**a**) The synthesis of Sm^III^-EC nanoparticles self-assembly. Reprinted with permission from reference [21], 2019 Wiley. (**b**) The synthesis of MPCP colloidal spheres. (**c**) SEM image of Co-Fe-TA colloidal spheres. Reprinted with permission from reference [27], 2018 Wiley. (**d**) The synthesis of iron-polyphenol colloidal nanoparticles. Reprinted with permission from reference [29], 2021 Elsevier.

MPCP nanoparticles have a large specific surface area and are easy to be functionalized, which have potential application value for the biomedical field [21,22,23,24]. The most convenient fabrication method of MPCP nanoparticles is the one-pot method. Polyphenols or their derivatives are simply mixed with different metal ions, while their coordination assembly can be completed spontaneously to obtain MPCP nanoparticles. For example, Li et al. prepared Sm^III^-EC nanoparticles for the treatment of colon cancer using the one-pot method [21]. During the preparation process, samarium ion (Sm^3+^) and polyphenol (-)-epicatechin (EC) were directly mixed and stirred at neutral pH for 24 h. Finally, Sm^III^-EC nanoparticles with a particle size of about 50 nm were obtained (Figure 3a). Sm^III^-EC NPs could accumulate in the tumor region and induce tumor cell apoptosis by damaging mitochondria. Meanwhile, Sm^III^-EGCG assembled by Sm^3+^ and (-)-epigallocatechin-3-gallate (EGCG) were prepared using the same method [22]. In addition, some anti-cancer drugs with phenolic hydroxyl structures can also participate in the coordination assembly to construct multifunctional MPCP NPs. For example, the AQ4N-Cu(II)-gossypol nanoparticles for cancer synergistic chemotherapy were reported by Wu’s group. The nanoparticles were synthesized by co-coordination of AQ4N (hypoxia-targeted chemotherapeutic drug) and gossypol with CuCl_2_ in ethanol solution (pH = 7.4) within 6 h [23].

Additionally, polyvinylpyrrolidone (PVP) was used as the surface coating agent to enhance the stability of MPCPs nanoparticles [24,25,26]. For instance, Liu et al. prepared Fe-ellagic acid (GA) nanodots with PVP protection [24]. In the preparation process, PVP and Fe^3+^ were firstly mixed to form PVP-Fe^3+^ complex, and then GA was introduced to the aqueous dispersion PVP-Fe^3+^ to form polymer nanodots (Fe-CPNDs). PVP played a regulatory and protective role in the nucleation and growth of Fe-CPNDs through the coordination of amide groups and Fe^3+^ ions. Fe-CPNDs with hydrated particle size of 5.3 nm and an electrically neutral surface, exhibited excellent photothermal properties and good colloidal stability. Moreover, Wei et al. developed a general sol–gel synthesis of MPCP colloidal spheres with a particle size of 300 nm and adjustable composition [27]. Tannic acid (TA), formaldehyde, and poloxamer F127 were precrosslinked into oligomer (PTA) under weak basic conditions. Then metal ions were added to form MPCPs through coordination with polyphenols. After heat treatment, MPCP colloidal spheres were successfully synthesized (Figure 3b,c). By adjusting the type and proportion of metal precursors, the MPCP colloidal spheres with single metal, bimetal, and multimetal species have been synthesized. Afterward, they further enhanced the approach to synthesize MPCP colloidal nanoparticles with an ultrasmall particle size (~20 nm) and exceptional colloidal stability [28,29,30]. During the preparation process, formaldehyde and TA were initially covalently crosslinked to form PTA polymers with small particle size, which effectively prevented the oxidation polymerization of TA. Metal ions were then added for coordination with TA to form MPCP. PVP was further utilized to cover the surface of MPCPs, which effectively prevented their aggregation and ultimately obtained ultrasmall MPCP colloidal nanoparticles with high colloidal stability. The synthesis strategy exhibited excellent universality and enabled the synthesis of a variety of MPCP nanoparticles, including Gd-TA, Mn-TA, Cu-TA, Ni-TA, Zn-TA, and Fe-TA. Furthermore, this approach facilitated precise control over both size and composition of MPCPs (Figure 3d).

#### 2.1.2. MPCP Crystal Particles

**Figure 4:**
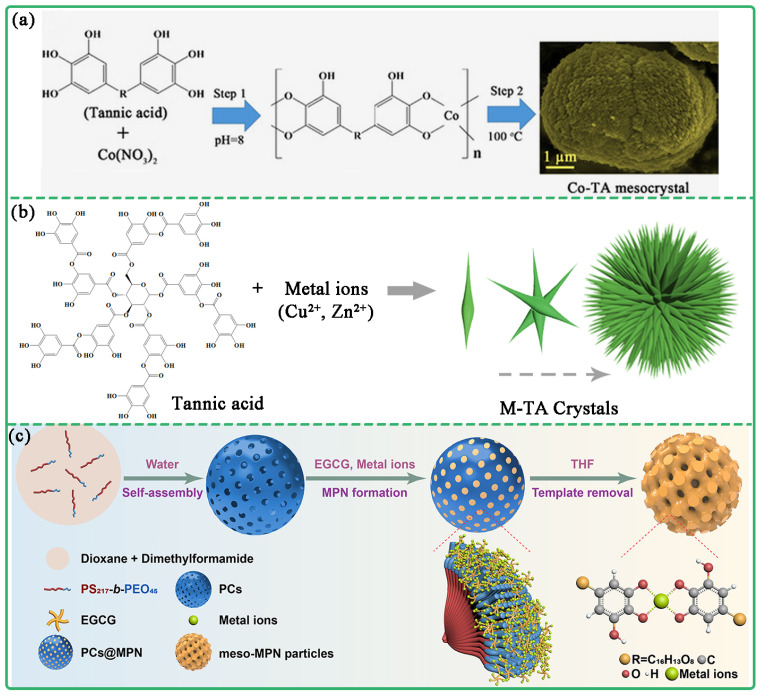
(**a**) Synthesis diagram and SEM image of Co-TA crystal particles. Reprinted with permission from reference [31], copyright 2016 Elsevier. (**b**) Schematic diagram of synthesis of Cu-TA crystals in the form of sea urchins. Reprinted with permission from reference [32], copyright 2018 Royal Society of Chemistry. (**c**) Schematic diagram of preparing mesoporous MPCP particles using the template method. Reprinted with permission from reference [34], copyright 2020 American Chemical Society.

Metal-organic frameworks (MOFs) are mostly constructed through the coordination of metal and organic ligand such as terephthalic acid. However, the majority of MOFs exhibit crystal structures, with only a minority employing polyphenols as ligands. Conversely, most MPCPS are amorphous structures, and very few are crystalline [31,32,33]. Wei et al. prepared Co-TA crystals using the hydrothermal method [31]. Co^2+^ and TA were first self-assembled to form Co-TA complex under alkaline conditions. Co-TA crystals were then further obtained via hydrothermal treatment. During hydrothermal treatment, TA ligands were oxidized and polymerized, resulting in the rearrangement MPCP (Figure 4a). In addition, Wei et al. prepared MPCP crystals with sea-urchin-like structures based on the self-assembly of metal and polyphenols [32]. Poloxamer F127 and TA molecules were dissolved in alkaline water/alcohol solvent. Then, Cu^2+^ was added to crosslink TA. After hydrothermal treatment, the Cu-TA MPCP crystals were obtained. The coordination assembly of metal ions and TA was modulated by changing the amount of ammonia, as pH exerted an influence on the process. As the amount of ammonia increased, the shuttle-like Cu-TA crystals gradually changed into urchin-like structures (Figure 4b). In addition, Zhao et al. prepared Fe-EA crystal particles with rhombohedral structure for magnetic resonance imaging and photothermal treatment [33]. FeCl_3_, ellagic acid (EA) and PVP solution were mixed and stirred, and then hydrothermal treatment was carried out to obtain Fe-EA crystal particles. It was found that the molecular weight of PVP affected the shape of the MPCP crystal, and the amount of PVP affected the size of the MPCP crystal. Fe-EA crystal particles showed good magnetic resonance imaging properties and effectively ablated tumors via photothermal therapy.

#### 2.1.3. Mesoporous MPCP Particles

Mesoporous MPCP particles are usually prepared using the template method. For example, Caruso et al. prepared mesoporous MPCP by sacrificing the block copolymer template [34]. The polystylene-polyoxyethylene (PS-b-PEO) was first assembled into a network polymer cube, and then EGCG was injected into the void and coordinated with Fe^3+^ to assemble EGCG-Fe(III) MPCPs. Finally, the polymer cube template was etched using tetrahydrofuran (THF) to produce mesoporous EGCG-Fe(III) MPCPs (Figure 4c). The porous structure of EGCG-Fe(III) MPCPs (≈40 nm) provides sufficient space for loading biomolecules such as bovine hemoglobin and glucose oxidase.

### 2.2. MPCP Capsules

Metal ions and polyphenols can be self-assembled on the template to form hollow MPCP capsules. MPCP capsules can loaded small molecules of drugs and biomolecules, thereby improving the stability and biocompatibility of these substances. Common templates include SiO_2_ microspheres, CaCO_3_ particles, and emulsion droplets, etc. Ethanol, hydrofluoric acid, and ethylenediaminetetraacetic acid (EDTA) can be used as scavengers. Spherical, linear, or sheet MPCP capsules can be prepared based on different shaped templates, and the thickness of the capsules can be controlled by optimizing the reaction conditions [35].

**Figure 5:**
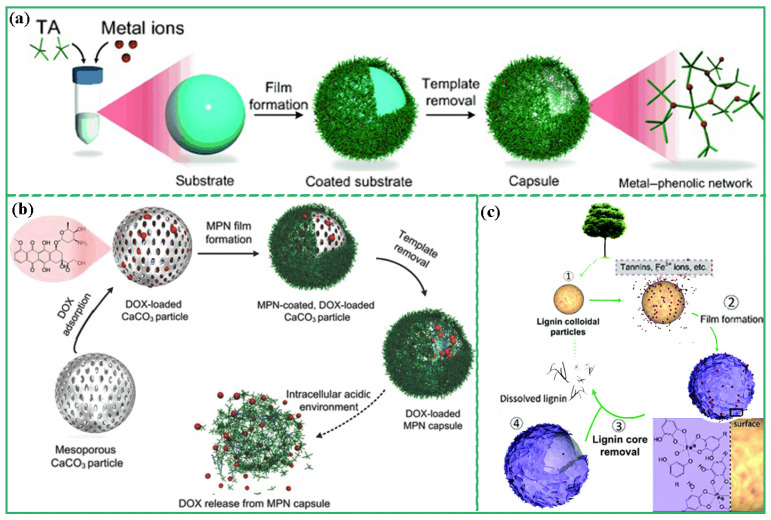
(**a**) Schematic illustrations of synthesized MPCP capsules using PS microspheres as templates. Reprinted with permission from reference [35], copyright 2014 Wiley. (**b**) Schematic illustrations of preparation of Fe-TA capsules using lignin as templates. Reprinted with permission from reference [36], copyright 2015 Wiley. (**c**) Schematic illustration of the fabrication process of DOX-loaded MPCP capsules and release mechanism of DOX from MPCP capsules. Reprinted with permission from reference [37], copyright 2018 Royal Society of Chemistry.

According to the properties of the template, MPCP capsule preparation methods can be divided into the hard template method and soft template method. For example, Caruso et al. fabricated a multifunctional MPCPs capsule library assembled with TA and various metal ions by utilizing polystyrene microspheres (PC) as templates (Figure 5a) [35]. The properties of MPCPs capsules were determined by the coordination metal ions, which had potential applications in drug delivery, imaging, and disease treatment. CaCO_3_ particles are also one of the widely used templates for the preparation of MPCP capsules because of their simplicity in synthesis, ease of size and shape control, and mild conditions for template removal. Ping et al. prepared a DOX-loaded Al^III^-TA MPCP capsule using the template method [36]. Polystyrene sulfonate (PSS)-doped CaCO_3_ templates were first prepared via the coprecipitation method. Subsequently, DOX was loaded into the CaCO_3_ particles, followed by the coordination of Al^3+^ and TA on the surface of CaCO_3_ to form Al^III^-TA MPCPs. After the template was removed via EDTA, DOX-loaded Al^III^-TA MPCP capsules with pH response were finally obtained (Figure 5b). Tardy et al. proposed a method for the preparation of MPCP capsules using lignin as a renewable and easily degradable template [37]. Initially, lignin particles were prepared in an aerosol flow reactor. Subsequently, Fe^3+^ and TA solutions were introduced into the colloidal suspension of the lignin particles, and Fe(III)-TA MPCPs were assembled on the surface of the lignin particles. Finally, sodium phosphate was employed to eliminate the template and obtain functional MPCP capsules (Figure 5c).

Emulsions are considered to be an excellent template for molecular self-assembly. Because the emulsion consists of two insoluble liquid bodies (such as water and oil), both hydrophobic and hydrophilic substances can be dissolved in the emulsion [38]. After mixing polyphenols and metal ions in the emulsion, MPCPs can self-assemble at the liquid-liquid interface or within the emulsion. Besford et al. reported a strategy to synthesize pH-responsive MPCPS at the emulsion interface [39]. Firstly, oleic acid emulsion with a diameter of 100–250 nm was prepared via the ultrasonic method. Polyphenol ligands modified with poly (ethylene glycol) (PEG) and metal ions were assembled at the emulsion interface to form MPCPs. The addition of PEG forms a protective barrier on the emulsion phase, effectively reducing fouling. Alternatively, the MPCP can self-assemble within the emulsion. For example, Dai et al. prepared PtP NPs by the coordination of Pt prodrug, PEG-modified polyphenol, and Fe^3+^ in emulsion [40]. Firstly, Pt prodrug polyphenols and PEG-polyphenols were mixed with FeCl_3_ solution. Because the stoichiometric ratio of Fe^3+^-polyphenol ligand was controlled by pH, FeCl_3_ solution is acidic, which led to the formation of Fe-polyphenol monocomplex. The oil phase (n-hexane, Triton X-100, and n-hexyl alcohol) was then mixed with a monocomplex solution to form an oil-in-water emulsion. The pH was then increased by adding Tris buffer (pH 8.5) to promote the formation of the stable Fe-polyphenol triscomplex. PtP NPs with 100 nm were obtained by removing the emulsion template via ethanol washing.

### 2.3. MPCP Films and Coatings

MPCP films and coatings can give special properties and functions to the substrate, so as to achieve functional modification of the substrate. Due to the extensive adhesion, polyphenols can be attached to different substrates (such as graphene oxide, gold nanoparticles, nanodiamonds, and bacteria). By means of coordination crosslinking of polyphenols and metal ions on the substrate, MPCP nanofilms or coatings can be constructed. MPCP films or coatings can be prepared via deposition, spraying, and electrochemical assembly.

**Figure 6:**
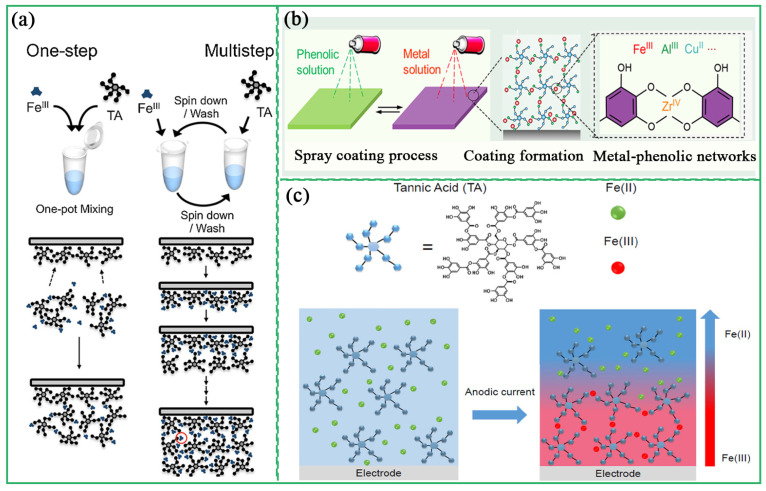
(**a**) Schematic illustrations of one-step and multistep assembly of Fe^2+^ and TA. Reprinted with permission from reference [41], 2013 American Association for the Advancement of Science. (**b**) Schematic illustrations of synthesized MPCP coating by spraying. Reprinted with permission from reference [43], 2018 American Chemical Society. (**c**) Schematic illustrations of MPCP coating prepared by electrochemical assembly. Reprinted with permission from reference [45], 2017 American Chemical Society.

The process of preparing MPCP films via the deposition method includes the one-step method and multistep method. In the one-step deposition assembly, polyphenol ligands and metal ions are directly mixed and assembled in a pot, followed by substrate immersion and subsequent deposition of the MPCP films onto its surface. Caruso et al. reported the synthesis strategy of Fe^III^-TA films assembled using a one-step method [41]. In this system, tannic acid and FeCl_3_ were directly mixed in water using polystyrene as substrates to form MPCP films (Figure 6a). Yun et al. prepared MPCP coatings with adjustable shape, thickness, and composition based on the deposition method [17]. For multistep deposition, excess polyphenols and metal ions are sequentially incubated onto the substrate. During the deposition process, unassembled polyphenol ligands and metal ions are eliminated to achieve a precisely controlled MPCP film (Figure 6a). In general, MPCP films prepared via multistep deposition have higher metal content, and MPCP films prepared via one-step deposition have a higher Young’s modulus [42].

MPCP films prepared using the spray method are less time-consuming and the physical and chemical properties of the films are easier to control [43,44]. Zhong et al. prepared MPCP films using the spray method (Figure 6b) [43]. The physicochemical properties (thickness, roughness, and major coordination states) of the MPCP were precisely controlled by adjusting the pH, the concentration, and the molar ratio of the polyphenol ligands to the metal ions. The electrochemical assembly method can also be used to prepare MPCP coatings, which is convenient and does not require complicated operation procedures. Maerten et al. proposed a strategy to prepare Fe^III^-TA nanocoatings via electrotriggered confined self-assembly [45]. In this system, Fe^II^ was oxidized to Fe^III^ using anode current, and Fe^III^-TA coatings were formed via local self-assembly near the electrode (Figure 6c). The thickness of MPCP coatings was controlled by switching the power supply, adjusting current intensity and action time.

**Table 1:** Structures and fabrication methods of representative MPCPs.

MPCPs	Metal Ions	Polyphenols	**Structure**	Size (nm)	Fabrication Method	Ref.
Sm^III^-EC	Sm^3+^	EC	nanoparticle	~50	one-pot method	[21]
Sm^III^-EGCG	Sm^3+^	EGCG	nanoparticle	~61	one-pot method	[22]
AQ4N-Cu(II)-gossypol	Cu^2+^	gossypol	nanoparticle	~88	one-pot method	[23]
Fe-CPNDs	Fe^3+^	GA	nanoparticle	~5	self-assembly with PVP assistance	[24]
FGPN	Fe^3+^	GA	nanoparticle	~6	self-assembly with PVP assistance	[25]
Fe-GA-PEG CPNs	Fe^3+^	GA	nanoparticle	~20	self-assembly with PVP assistance	[26]
Zn-TA	Zn^2+^	TA	colloidal sphere	~300	sol–gel synthesis	[27]
Gd-TA	Gd^3+^	TA	colloidal nanoparticle	~21	sol–gel synthesis	[28]
Fe-TA	Fe^2+^	TA	colloidal nanoparticle	~25	sol–gel synthesis	[29]
Gd/Fe-TA	Gd^3+^/Fe^2+^	TA	colloidal nanoparticle	~23	Sol–gel synthesis	[30]
Co-TA	Co^2+^	TA	crystal particle	~5000	hydrothermal method	[31]
Cu-TA	Cu^2+^	TA	crystal particle	~3000	hydrothermal method	[32]
Fe-EA	Fe^3+^	EA	crystal particle	~240	self-assembly with PVP assistance	[33]
EGCG-Fe(III)	Fe^3+^	EGCG	mesoporous particles	~2400	hard template-based self-assembly	[34]
Al^III^-TA	Al^3+^	TA	capsule	~2500	hard template-based self-assembly	[36]
Fe(III)-TA	Fe^3+^	TA	capsule	~330	hard template-based self-assembly	[37]
E-MPNs	Fe^3+^	PEG-polyphenol	capsule	100–250	emulsion template-based self-assembly	[39]
PtP NPs	Fe^3+^	PEG-polyphenol	capsule	~100	emulsion template-based self-assembly	[40]
Fe^III^-TA	Fe^3+^	TA	Film/coating	/	deposition	[41]
Fe(III)-TA	Fe^3+^	TA	Film/coating	/	spray method	[44]
TA-Fe(III)	Fe^3+^	TA	Film/coating	/	electrotriggered self-assembly	[45]

## 3. Biomedical Applications of MPCPs

The fabrication process of MPCPs is facile and eco-friendly [46,47]. Via functionalization, MPCPs can be engineered into multifunctional nanoplatforms that augment therapeutic efficacy against diseases [48,49,50]. Additionally, the pH responsiveness of the MPCPs enables targeted drug delivery and controlled release, thereby enabling precise disease treatment [51,52,53,54,55]. Moreover, the biocompatibility profile of MPCPs is excellent with negligible toxicity [56,57,58]. MPCPs have been extensively investigated in the field of biomedicine such as biosensing, drug delivery, bioimaging, tumor therapy, and antibacterial applications (Table 2) [59,60,61,62,63].

### 3.1. Biosensing

**Figure 7:**
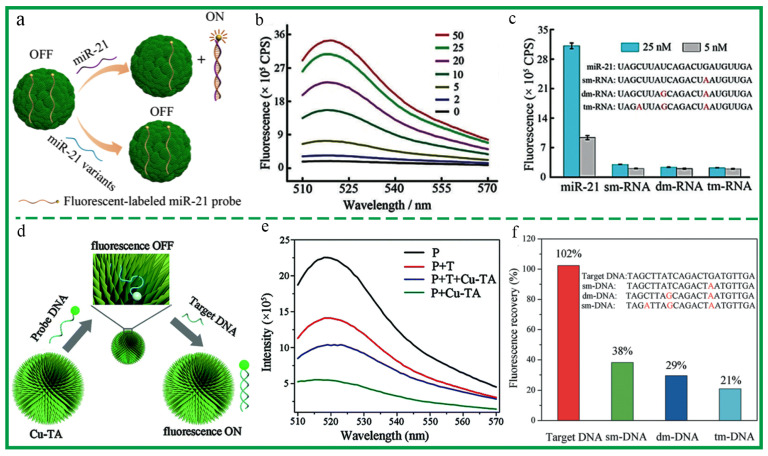
(**a**) Schematic diagram of MPCP colloidal spheres based on fluorescent DNA detection. (**b**) Fluorescence recovery in different concentrations of miR-21. (**c**) Selectivity for the detection of miR-21. Reprinted with permission from reference [27], 2018 Wiley. (**d**) Schematic diagram of Cu-TA crystals based on fluorescent DNA detection. (**e**) Fluorescence spectra of the probe DNA under different conditions: (**f**) Selectivity for the detection of the DNA. Reprinted with permission from reference [32], 2018 Royal Society of Chemistry.

Biosensor can convert physical and chemical information into measurable data by attaching targeting or detection ligands (aptamers and antibodies) to nanomaterials, which has enormous potential in the early diagnosis of diseases. Wei et al. integrated MPCP colloidal spheres with fluorescent probe nucleic acid (ssDNA) to construct a detection platform for nucleic acid [27]. As the quenchers, MPCP colloidal spheres could adsorb probe ssDNA and quench its fluorescence through π-π accumulation or metal ions coordination with probe ssDNA. In the presence of target-ssDNA, probe ssDNA and target-ssDNA could hybridize into a double-stranded nucleic acid structure through base complementary pairing. The probe ssDNA was desorbed from the surface of MPCP colloidal spheres and its fluorescence was restored (Figure 7a). Based on the fluorescence intensity, the concentration of the target-ssDNA (tumor marker miRNA-21) could be quantitatively analyzed with high sensitivity. Furthermore, the nucleic acid platform demonstrated exceptional selectivity and proficiently distinguished single-base discrimination (Figure 7b,c). Meanwhile, Wei et al. also built a highly efficient, sensitive, and selective nucleic acid sensing platform based on Cu-TA crystals [32]. The nanoplatform realized the quantitative detection of tumor marker miRNA-21 in the concentration range of 0–10 nM (Figure 7d,e). Moreover, the nanoplatform exhibited high selectivity in discriminating target DNA from those with single-, double-, and triple-base mismatches (Figure 7f).

### 3.2. Drug Delivery

**Figure 8:**
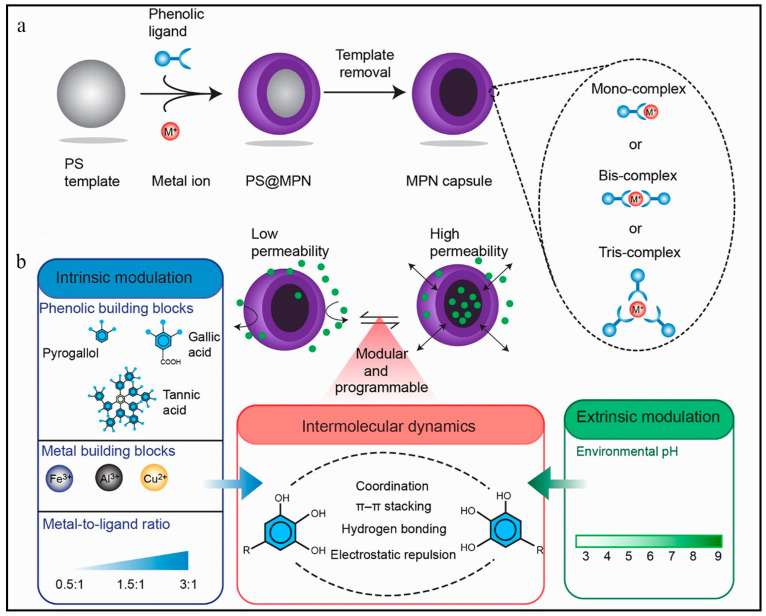
(**a**) Schematic illustrations of an MPCP capsules formed on a PS template particle. (**b**) Schematic illustrations of programming the permeability of MPCP capsules by endogenous and exogenous regulation of intermolecular dynamics. Reprinted with permission from reference [65] Copyright 2020 Wiley.

Drug delivery systems can deliver therapeutic drugs to target tissues, increase the utilization rate of drugs, and reduce the side effects of drugs [36,37,38,39]. With excellent biocompatibility and external stimulus responsiveness, MPCPs have been used to construct ideal drug delivery systems [49,50,51,52,53,54,55,64,65,66,67]. For example, Caruso et al. constructed MPCP capsules with a dynamic gating mechanism by adjusting the intermolecular interactions. The MPCP capsules responded at pH values between 4 and 9 to achieve “off” and “on” state transitions and accurately controlled the dynamic encapsulation and release of drugs (Figure 8) [65]. For MPCP-based drug delivery systems, small molecules are typically loaded into hollow nanoparticles, which are further protected by MPCP coatings. For example, DOX molecules were first loaded in mesoporous silica, and then the Fe(III)-TA coatings were modified [66]. The MPCP coatings served as a “gatekeeper” of pH response, releasing drugs on demand. Li et al. fabricated Cu-TA that functionalized onto the surface of mesoporous silica to prevent the premature drug (DOX) release [67]. In the acidic environment of a tumor, the MPCP shells degraded and DOX molecules were released in targeted locations. In addition, small molecule drugs and MPCP can be assembled into particles to realize drug delivery. Dai et al. constructed a drug delivery platform based on the coordination assembly of DOX, Fe^3+^, Pt-prodrug polyphenol, and PEG-polyphenol [47]. The nanoparticles accumulated in the tumor site for 72 h, and the tumor growth was effectively inhibited by the synergistic treatment of DOX and platinum drugs.

### 3.3. Bioimaging

Bioimaging plays an important role in all stages of tumor screening, diagnosis, and the evaluation of curative effect [61,62,63,64,65,66,67,68,69]. MPCPs can be used as a nanocontrast agent for biological imaging, which overcome the shortcomings of high cytotoxicity and poor biological stability of traditional small molecular contrast agents [70].

#### 3.3.1. Magnetic Resonance Imaging (MRI)

**Figure 9:**
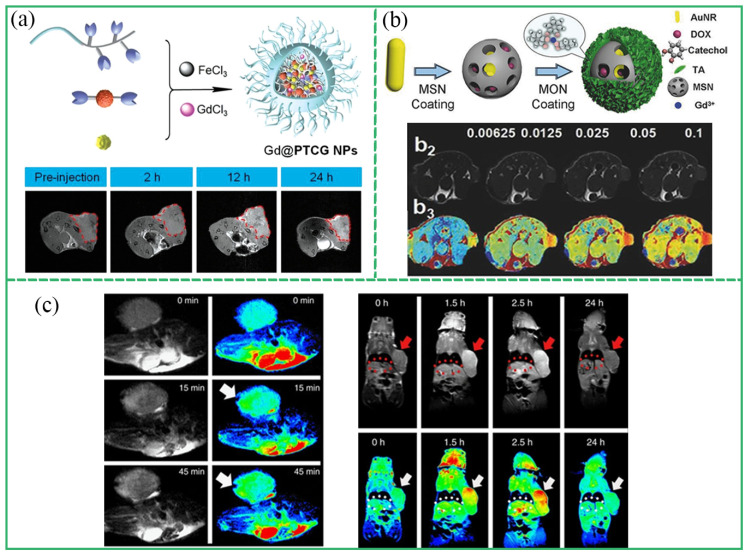
(**a**) Schematic diagram of preparation of Gd@PTCG NPs and MRI of tumors at different time. Reprinted with permission from reference [72], 2020 Wiley. (**b**) Schematic diagram of the preparation process of TA-Gd(III) and MRI of tumors with different concentration of TA-Gd(III). Reprinted with permission from reference [73], 2017 Wiley. (**c**) MRI of intratumor injection of Fe-CPNDs (tumor and arrow). Reprinted with permission from reference [24], 2015 Springer Nature.

MRI has the characteristics of noninvasive, nonradiation, and high spatial resolution, and has become one of the most effective diagnostic methods for cancer and cardiovascular diseases. MR contrast agents can enhance the MRI signal by shortening the relaxation time of water protons [71]. The traditional MR contrast agents have the potential risk of a low relaxation rate and metal ion leakage. The development of new nanocontrast agents has greatly improved the performance of MRIs. MPCPs with coordination of polyphenols and metal ions such as Gd^3+^, Mn^2+^, and Fe^3+^ have been used as MR contrast agent for biological imaging in vivo. For example, EGCG, Gd^3+^, Fe^3+^, and platinum prodrugs (Pt-OH) were assembled into a multifunctional nanoplatform (Gd@PTCG NPs) for image-guided therapy (Figure 9a) [72]. Gd@PTCG NPs showed good biocompatibility, a high longitudinal relaxation rate (*r*_1_ = 4.95 ± 0.44 mM^−1^s^−1^), and low risk of leakage of Gd^3+^. Gd@PTCG NPs, as a *T*_1_-weighted magnetic resonance contrast agent, significantly enhanced the MR signal of the tumor site in mice. Meanwhile, the NPs improved treatment efficacy through MRI-guided precision chemotherapy. Fan et al. constructed a multifunctional nanoplatform (AuNR@MSN@MON) by coating Gd-TA on mesoporous silica (MSN) containing gold nanorods and DOX (Figure 9b) [73]. As a *T*_1_-weighted magnetic resonance contrast agent, the nanoplatform enhanced the MR signal of tumor tissue in mice. Moreover, the distribution and metabolism of nanomaterials in major organs and tumors of mice were evaluated using MRI. A total of 2.5 h after the injection of Fe-CPNDs nanoparticles, the MR signal of the tumor site reached the maximum, indicating that Fe-CPND was effectively accumulated in the tumor through an enhanced permeability and retention (EPR) effect (Figure 9c) [24]. Zhao et al. built a multifunctional nanoplatform based on Fe-EA NPs [33]. In the study, Fe-EA NPs showed good *T*_2_-weighted MRI ability (*r*_2_ = 24.62 mM^−1^s^−1^), guiding efficient photothermal therapy and enabling integrated tumor diagnosis and treatment.

#### 3.3.2. Photoacoustic Imaging (PAI)

PAI is a living biological imaging technology with high spatial resolution. Under laser action, biological tissue absorbs light energy and subsequently generates ultrasonic waves, which are then detected and utilized for the reconstruction of photoacoustic images [74]. A near-infrared laser is usually used as the source of PAI due to its strong tissue penetration. In recent years, MPCPs have been widely studied as photoacoustic contrast agents. For example, Guo et al. coated a Mn-TA network on black phosphorus (BPNS) nanosheets to prepare multifunctional-nanocomposite BPNS@TA-Mn. Under the irradiation of a near-infrared 808 nm laser, Mn-TA showed good photoacoustic imaging performance and guided BPNS to realize efficient photothermal therapy [75]. Li et al. prepared a nanoplatform with multimodal imaging and combination therapy by coating EGCG-Fe on mesoporous silica nanoparticles [76]. EGCG-Fe coating exhibited excellent photothermal conversion efficiency (*η* = 47.7%) and photoacoustic imaging performance.

#### 3.3.3. Positron Emission Tomography (PET)

PET is a highly sensitive medical imaging technology, which can show brain functional activities and metabolic changes in vivo. MPCPs assembled using metal isotopes and polyphenols have been widely used in PET imaging. Wang et al. prepared supramolecular MPCP nanoparticles assembled by ^89^Zr, TA and poloxamer F127, and fluorescent dyes (IR780) for near-infrared fluorescence imaging (NIRF) and PET dual-mode imaging in mice [77]. ^89^Zr-TA exhibited high stability in mouse blood, and the leakage rate was less than 3% within 72 h. PET in vivo monitors the dynamic distribution of nanoparticles in mice, providing accurate information on particle accumulation and liver clearance in tumor areas. Additionally, Jin et al. prepared ^64^Cu-labeled Fe-GA-PEG CPNs multifunctional nanoparticles for whole-body PET imaging in mice [26]. The biological distribution of Fe-GA-PEG CPNs in vivo and the enrichment of tumor areas were successfully revealed by PET imaging.

#### 3.3.4. Other Imaging Modes

X-ray computed tomography (CT) provides high-resolution three-dimensional image information, which is a widely used imaging technology in clinical diagnosis. Li et al. constructed a multifunctional nanocomposite for three-mode CT/MRI/luminescence imaging based on BaGdF_5_ nanoparticles and TA-Eu^3+^ coating [78]. The CT performance of composites was attributed to the strong absorption of X-rays by atoms with high atomic numbers, and CT imaging reflected the comprehensive information of deep tissues in mice. Fluorescence imaging is widely used in cell tracking and organ/tissue imaging in biomedicine. In recent years, MPCPs as fluorescent probes have been reported. For example, Caruso et al. prepared Tb^III^-TA and Eu^III^-TA capsules, which were used to show green (545 nm) and red (613 nm) fluorescence [35]. At the same time, acetylacetone (AA) and 2-thenoyltrifluoroacetone (TTA) were introduced as coligands to enhance the fluorescence intensity of Tb^III^-TA and Eu^III^-TA MPCP capsules.

Ultrasound imaging is a basic diagnostic technique, which can track blood flow rate and examine pathological tissues through high-resolution imaging. The introduction of contrast agent can obviously improve the ultrasonic imaging effect. Fe-TA capsules could change acoustic impedance to produce specific nonlinear oscillations and realize real-time detection of tumor tissues through ultrasonic imaging [79].

### 3.4. Tumor Therapy

With the development of nanomedical technology, new tumor treatment methods have emerged, such as photothermal therapy, chemodynamic therapy, photodynamic therapy, and so on [17,18,19,20]. Generally, emerging tumor treatment methods need nanomaterials to achieve good therapeutic effects, and MPCPs are considered as the ideal nanotreatment platform [17,18,19,20].

#### 3.4.1. Chemotherapy

**Figure 10:**
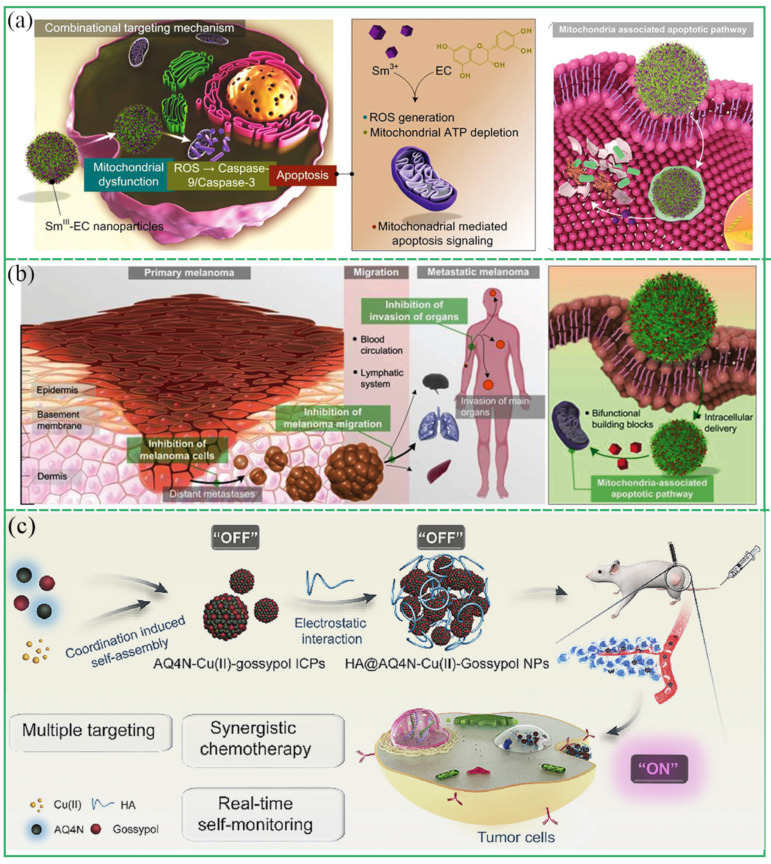
(**a**) Pathways and mechanisms of Sm^III^-EC nanoparticles enucleated by tumor cells to induce mitochondrial apoptosis. Reprinted with permission from reference [21], 2019 Wiley. (**b**) Schematic diagram of the development of metastatic melanoma and mechanism of apoptosis induced by Sm^III^-EGCG nanocomplex. Reprinted with permission from reference [22], 2019 Elsevier. (**c**) Schematic illustration of HA@AQ4N-Cu(II)-gossypol for efficient synergistic chemotherapy. Reprinted with permission from reference [23], 2018 Elsevier.

Chemotherapy is a traditional and important cancer treatment method. Small molecular chemotherapy drugs are easily eliminated in the body, and lack of targeting to the focus, which easily causes systemic toxicity [20]. Polyphenols have shown great potential in chemotherapy in recent years. Polyphenols such as TA, EGCG, and Gossypol have strong antioxidant activity and the ability to scavenge reactive oxygen species (ROS). As the ideal nanotherapeutic drug, MPCPs have broad application prospects [80,81,82]. For example, Li et al. constructed a nanosystem for targeted cancer therapy based on Sm^III^-EC NPs [21]. Sm^III^-EC NPs were internalized by tumor cells and interpreted to release Sm^3+^ and EC therapeutic modules under acidic conditions. ROS production was enhanced by changing mitochondria-related pathways in cells, thereby inducing tumor cell apoptosis (Figure 10a). Similarly, a tumor chemotherapy platform based on Sm^III^-EGCG NPs was reported by Li et al., which induced apoptosis of cancer cells by changing the metabolic pathway of melanoma cells (Figure 10b) [22]. In addition, some anticancer drugs and polyphenols as double ligands can coordinate with metal ions to construct multifunctional MPCP nanoplatforms for highly efficient treatment of cancer. Wu’s group prepared the chemotherapeutic drug AQ4N-Cu(II)-gossypol NPs based on the co-ordination of AQ4N and gossypol with CuCl_2_ [23]. AQ4N-Cu(II)-gossypol NPs exhibited multiple tumor-targeting abilities attributed to the CD44 receptor, pH responsiveness, and AQ4N hypoxia. AQ4N not only cooperated with gossypol to achieve highly efficient cancer synergistic chemotherapy with tumor inhibitory rates of 87% but also monitored realtime drug release and distributions in vivo (Figure 10c).

#### 3.4.2. Photothermal Therapy (PTT)

**Figure 11:**
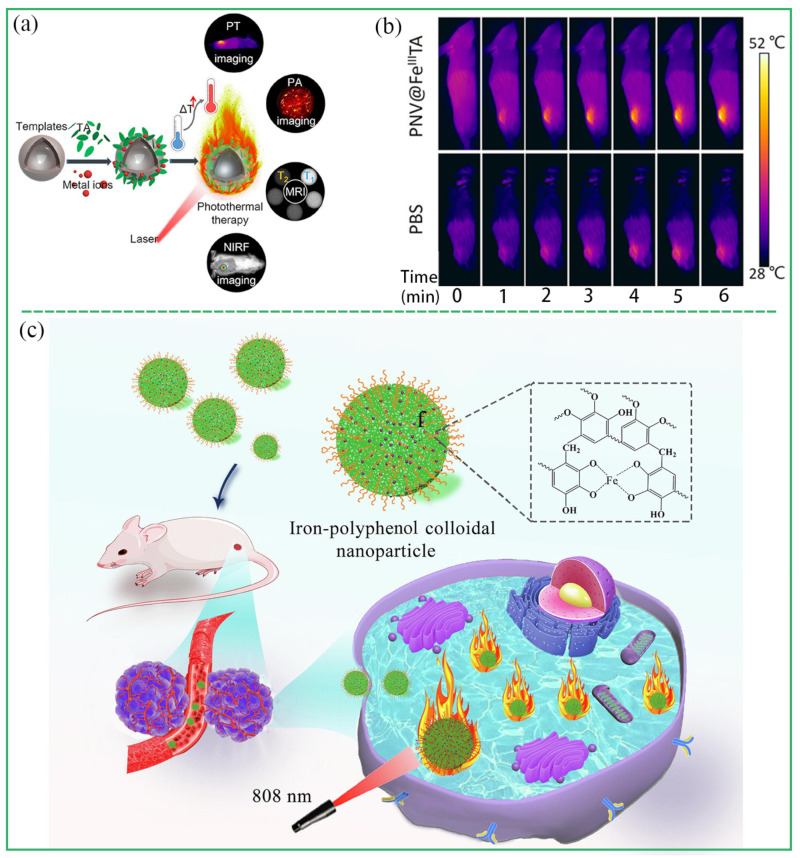
(**a**,**b**) Template preparation of PNV@FeIII-TA and its PTT application for tumor therapy. Reprinted with permission from reference [84] 2018 American Chemical Society. (**c**) Schematic illustration of PTT applications of Fe-TA colloidal nanoparticles. Reprinted with permission from reference [29] 2021 Elsevier.

PTT has many advantages, such as high selectivity, less invasion, and less damage to normal tissues, and it has a broad application prospect in tumor treatment. The mechanism of PTT mainly includes two aspects: (1) A photothermal agent (PTA) selectively aggregates in tumor tissues; (2) PTA absorbs near-infrared light and converts it into local heat energy, thus killing tumor cells [83]. However, the problems of poor biological safety, low photothermal conversion efficiency, and the complex synthesis process of traditional PTAs limit their wide application. MPCPs with iron-polyphenol are an ideal PTA because of the excellent photothermal conversion efficiency, good photothermal stability, and biocompatibility [84,85,86,87,88,89]. For example, Feng et al. prepared a variety of MPCPs based on the coordination and assembly of TA with different metal ions, and systematically explored the effects of metals ions (Gd^III^, Ru^III^, Fe^III^, Cu^II^, Ni^II^, Mn^II^, and V^III^) on the photothermal properties of MPCPs [84]. Under the irradiation of 808 nm laser, the same concentration of MPCPs showed obvious differences in photothermal properties. Among them, Fe^III^-TA, V^III^-TA, and Ru^III^-TA showed good photothermal performance, while MPCP based on Gd^III^, Cu^II^, Ni^II^, and MnII had almost no photothermal effect. This result proved that the photothermal properties of MPCP have dependence upon metal ions (Figure 11a,b). Qin et al. synthesized Fe-TA nanoparticles exhibiting excellent photothermal conversion efficiency (*η* = 43.6%) and favorable biocompatibility, which effectively suppressed tumor growth through the photothermal effect with an inhibition rate of 93.2%. (Figure 11c) [29]. Zhao et al. prepared Fe-EA NPs to achieve photothermal treatment of tumors [33]. Because the charge transfer between ligand and metal ions had a wide absorption band in the near infrared region, it caused a strong photothermal effect. The photothermal effect of Fe-EA NPs effectively ablated tumor cells and inhibited tumor growth. Fe-CPDNs nanoparticles prepared by Liu et al. had excellent photothermal properties, and tumor cells were effectively ablated by photothermal therapy [24].

#### 3.4.3. Chemodynamic Therapy (CDT)

**Figure 12:**
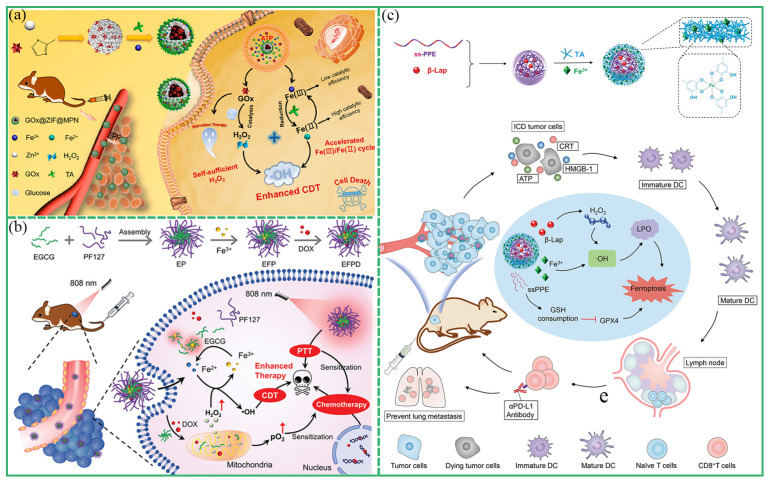
(**a**) Schematic diagram of the GOx@ZIF@MPCP-nanoparticle-catalyzed Fenton reaction used to enhance CDT and hunger therapy for tumor ablation. Reprinted with permission from reference [91], copyright 2018 American Chemical Society. (**b**) Schematic of the proposed self-enhanced synergistic PTT/CDT/chemotherapy mechanism mediated by EFPD. Reprinted with permission from reference [81], copyright 2023 Wiley. (**c**) Schematic illustration of the constructed ssPPELap@Fe-TA for amplified ferroptosis-synergized immunotherapy. Reprinted with permission from reference [103], copyright 2023 American Chemical Society.

CDT relies on the Fenton reaction to achieve its antitumor effect. The Fenton reaction generates ROS from a high concentration of H_2_O_2_ in the tumor microenvironment, resulting in the death of tumor cells [90]. In recent years, the antitumor research of MPCP based on CDT has been widely reported [91,92,93,94,95,96,97,98,99,100]. For example, Mao et al. prepared PTCG NPs based on Pt-OH, EGCG, and PEG-polyphenol for chemotherapy and chemodynamic therapy [72]. Through cascade reaction, the level of H_2_O_2_ was increased, and Fe^3+^ catalyzed H_2_O_2_ into toxic ROS, thus achieving an efficient CDT effect on tumors. In order to overcome the problems of low Fenton reaction efficiency and an insufficient H_2_O_2_ level in the tumor microenvironment, Zhang et al. reported that a self-catalyzed Fenton nano-system (GOx@ZIF@MPN) ablated tumor cells on the basis of CDT [91]. Glucose oxidase (GOx) catalyzed glucose to produce H_2_O_2_, which was further catalyzed by Fe^2+^ to produce highly toxic ·OH, thus achieving chemodynamic treatment of tumors (Figure 12a). Functionalized Fe-TA coated on NPs not only performed a Fenton reaction but also prevented GOx from premature leakage. In addition, Wu et al. reported self-amplified nanoreactors through the assembly of TA, Fe^3+^, and GOx [92]. The nanosystem consumed glucose to produce H_2_O_2_, which was further catalyzed and oxidized to highly toxic ·OH for efficient CDT [92]. Usually, CDT was used to combinate with PTT and chemotherapy to achieve optimal tumor treatment. Shi et al. designed a Fenton reaction amplifier through the assembly of EGCG, poloxamer F127, Fe^3+^, and DOX for the enhancement of synergistic PTT/CDT/chemotherapy [81]. The nanoplatforms inhibited cell respiration to reduce O_2_ consumption, thus boosting H_2_O_2_ generation for enhanced CDT and simultaneously improving DOX chemotherapy efficacy (Figure 12b).

#### 3.4.4. Other Treatments

The aim of tumor immunotherapy is to achieve tumor treatment through the stress immune response. Polyphenol itself can inhibit tumor growth through immunomodulation [4]. In recent years, the application of MPCP to regulate the immune system and activate immune response to kill tumors has been widely studied [101,102,103,104,105]. Wang et al. constructed a vaccine for immunotherapy via in situ assembly of an EGCG-Al(III) MPCP coating on living cells [101]. In this study, these nanoparticles protected the antigen from degradation in vivo, improved the efficiency of antigen uptake by cells, and delayed the retention time of antigen in lymph nodes. In addition, microparticles could trigger the activation of dendritic cells and stimulate the upregulate th1-related cytokines. Finally, the efficient individualized treatment of the tumor was realized. Meanwhile, Dai et al. developed a ssPPELap@Fe-TA nanodevice with a Fe-TA shell and disulfide-containing polyphosphoester (ssPPE) core with β-lapachone loading [103]. The nanodevice induced a systemic antitumor immune response by promoting dendritic cell maturation and T-cell infiltration, and acts in concert with anti-PD-L1 antibodies to significantly inhibit 4T1 tumor growth (Figure 12c). Furthermore, photodynamic therapy (PDT) is a minimally invasive tumor treatment method. Photosensitizers absorb light with a specific wavelength and convert the surrounding oxygen into cytotoxic ROS [106]. The application of MPCP as a photosensitizer delivery carrier and for the enhancement of PDT has been reported. For example, Wei et al. reported the study of pH-responsive Fe-PEG-polyphenol capsules carrying photosensitizer hematoporphyrin monomethyl ether (HMME) to cancer cells [107]. Due to the targeting effect of folic acid, MPCP@HMMEs were selectively enriched in tumor areas. HMME was released in the acidic tumor environment and ROS was produced under 638 nm laser irradiation to induce tumor cell death, thereby achieving photodynamic therapy of the tumor.

### 3.5. Antibacterial Application

**Figure 13:**
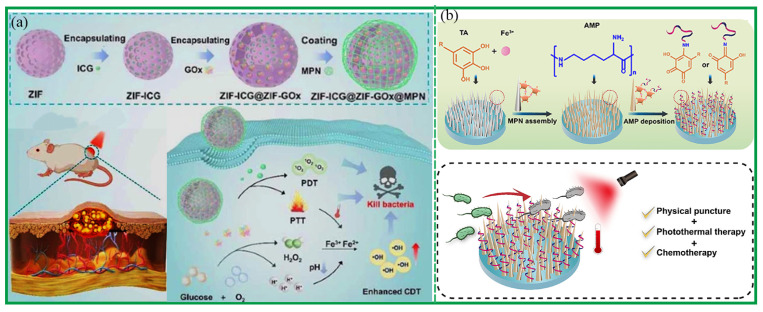
(**a**) Schematic illustration of the TNS-MPN-AMP nanocoating fabrication procedure [110]. Reprinted with permission from reference [110], copyright 2023 Elsevier. (**b**) Schematic illustration of the preparation process of ZIF-ICG@ZIF-GOx@MPN and its action mechanism for combined therapy to eradicate pathogenic bacteria. Reprinted with permission from reference [111], copyright 2023 American Chemical Society.

Polyphenols have excellent antioxidant activity and ROS scavenging activity. Metal ions with redox activity can induce bacterial oxidative stress and destroy bacterial cell walls. MPCPs have been reported to exhibit excellent antibacterial, antioxidant, and anti-inflammatory properties [108,109,110,111,112,113,114,115,116,117,118,119,120]. Li et al. prepared a multifunctional Cu^II^-TA coating with antioxidant, anti-inflammatory, antibacterial, and anticoagulant properties [108]. The residual phenolic hydroxyl group on the coating had antioxidant and anti-inflammatory activities, which endowed the coating with lasting and efficient antibacterial function. The application of MPCP-based photothermal therapy and photodynamic therapy is not limited to tumor treatment but also extends to antibacterial treatment. In general, in the applications of PTT and PDT to combat bacterial wound infection, increases in temperature and production of toxic ROS were limited to a manageable extent to prevent excessive damage to surrounding healthy tissues. [3]. Liao et al. constructed an antibacterial nanoreactor by encapsulating indocyanine green (ICG) and glucose oxidase (GOx) into ZIF-8 nanoparticles coated with Fe-TA [110]. The nanoreactors could combine CDT with PTT and PDT, which achieved high bactericidal efficiency (99.7% for MRSA) and alleviated the antibiotics resistance issues (Figure 13a). In addition, photothermal therapy was often used in combination with chemotherapy and physical therapy to enhance the antibacterial effect. Huo et al. constructed a multifaceted nanocoating via the hybridization of TiO_2_ nanospikes (TNSs), Fe-TA, and antimicrobial peptides (AMPs) [111]. The TNSs’ structure can disrupt the bacteria via physical puncture and Fe-TA proved excellent photothermal bactericidal properties. Furthermore, with the assistance of contact-active chemo bactericidal efficacy of AMPs, the nanocoating achieved physical/photothermal/chemo triple-synergistic therapy against pathogenic bacteria with a high antibacterial ratio of >99.99% in vitro (Figure 13b).

**Table 2:** Application of representative MPCPs.

MPCPs	Metal Ions	Polyphenols	Agent	Application	Ref.
Zn-TA	Zn^2+^	TA	—	biosensing	[27]
Cu-TA	Cu^2+^	TA	—	biosensing	[32]
Al^III^-TA	Al^3+^	TA	DOX	drug delivery	[36]
PS@TA/Fe^III^	Fe^3+^	TA	DOX	drug delivery	[65]
MSN@MPN	Fe^3+^/Al^3+^	TA	DOX	drug delivery	[66]
UCNP@MSN	Cu^2+^	TA	DOX	drug delivery	[67]
Gd@PTCG	Gd^3+^	EGCG	Pt-OH	MRI, chemotherapy	[72]
AuNR@MSN@MON	Gd^3+^	TA	DOX	MRI, chemotherapy	[73]
Fe-CPNDs	Fe^3+^	GA	—	MRI, PTT	[24]
^64^Cu-labeled Fe-GA-PEG CPNs	^64^Cu/Fe^3+^	GA	—	PET, MRI, PAT, PTT	[26]
Fe-EA NPs	Fe^3+^	TA	—	MRI, PTT	[33]
BPNS@TA-Mn	Mn^2+^	TA	black phosphorus	PAI, PTT	[75]
EGCG-Fe/PVP	Fe^3+^	FGCG	—	PAI, PTT	[76]
PPNPs	^89^Zr	TA	—	PET, NIRF	[77]
BaGdF5@MPN	Eu^3+^	TA	—	CT/MRI/luminescence imaging	[78]
Sm^III^-EC	Sm^3+^	EC	—	chemotherapy	[21]
Sm^III^-EGCG	Sm^3+^	EGCG	—	chemotherapy	[22]
AQ4N-Cu(II)-gossypol	Cu^2+^	gossypol	—	chemotherapy	[23]
Fe-CPNDs	Fe^3+^	GA		PTT	[24]
Gd/Fe-TA	Gd^3+^/Fe^2+^	TA		MRI, PTT	[30]
PTCG NPs	Fe^2+^/Fe^3+^	EGCG	Pt-OH	chemotherapy, CDT	[72]
EFPD	Fe^2+^/Fe^3+^	EGCG	DOX	PTT/CDT/chemotherapy	[81]
Fe^III^-TA	Fe^3+^	TA	—	PTT	[84]
GOx@ZIF@MPN	Fe^2+^/Fe^3+^	TA	GOx	CDT	[91]
ssPPE Lap@Fe-TA	Fe^3+^	TA	*β*-lapachone	immunotherapy	[103]
MPCP@HMMEs	Fe^2+^/Fe^3+^	PEG-polyphenol	—	PDT	[107]
MXene@EGCG	Fe^2+^/Fe^3+^	EGCG	Ti_3_C_2_Tx MXene	CDT, PTT, PDT	[110]

## 4. Conclusions

In recent decades, there has been a notable surge in the development of MPCPs for biomedical applications. The facile synthesis, adjustable structures, and excellent biocompatibility of MPCPs render them ideal for use in disease diagnosis and treatment. Based on MPCPs, an efficient biosensing platform can be constructed for the detection of biomolecules such as proteins and nucleic acids, providing advantages in early disease diagnosis and treatment. In particular, their multiresponsiveness enables the selective release of drugs or other therapeutic agents at disease sites while minimizing adverse effects on healthy tissues. Further modification of MPCPs with target molecules ensures superior biosecurity and efficient tumor enrichment, thereby achieving an enhanced theranostic effect. Additionally, MPCPs can integrate multimodal diagnosis and treatment, ultimately improving cancer treatment outcomes and enhancing antimicrobial efficacy. Overall, the exceptional properties of MPCPs have rendered them a highly sought-after topic in the biomedical field. This review has summarized the classification of various MPCP structures (particles, capsules, and coatings) and their production strategies. Then, the significant achievements of MPCPs in the biomedical field such as biosensing, drug delivery, bioimaging, antitumor, and antibacterial therapies are highlighted.

Despite the significant achievements that have been attained in MPCPs, there are still several perspectives for further improvement in future research: (1) MPCPs serve as therapeutic platforms; the function of polyphenol itself should be more fully utilized to enhance the therapeutic effect of tumor. (2) In order to improve the accuracy of tumor therapy, it is necessary to modify the MPCPs to target ligands that specifically bind to the receptors on the surface of cancer cells. (3) The side effects of tumor therapy cannot be ignored and the stability of MPCPs should be emphasized. MPCP carriers, pH-responsive nanomaterials, should release drugs at specific locations or conditions to avoid early drug leakage and damage to normal tissues. Finally, the review is expected to serve as a catalyst for further research into the biomedical applications of MPCP. Through structural and property optimization, MPCP has the potential to become an indispensable and highly effective nanomedicine in the field of biomedicine.

## Data Availability

Not applicable.
